# Evidence-Based Guidelines for Mental, Neurological, and Substance Use Disorders in Low- and Middle-Income Countries: Summary of WHO Recommendations

**DOI:** 10.1371/journal.pmed.1001122

**Published:** 2011-11-15

**Authors:** Tarun Dua, Corrado Barbui, Nicolas Clark, Alexandra Fleischmann, Vladimir Poznyak, Mark van Ommeren, M. Taghi Yasamy, Jose Luis Ayuso-Mateos, Gretchen L. Birbeck, Colin Drummond, Melvyn Freeman, Panteleimon Giannakopoulos, Itzhak Levav, Isidore S. Obot, Olayinka Omigbodun, Vikram Patel, Michael Phillips, Martin Prince, Afarin Rahimi-Movaghar, Atif Rahman, Josemir W. Sander, John B. Saunders, Chiara Servili, Thara Rangaswamy, Jürgen Unützer, Peter Ventevogel, Lakshmi Vijayakumar, Graham Thornicroft, Shekhar Saxena

**Affiliations:** 1 Department of Mental Health and Substance Abuse, World Health Organization (WHO), Geneva, Switzerland; 2 WHO Collaborating Centre for Research and Training in Mental Health, Department of Public Health and Community Medicine, Section of Psychiatry and Clinical Psychology, University of Verona, Verona, Italy; 3 Universidad Autonoma de Madrid, CIBERSAM, Madrid, Spain; 4 International Neurologic and Psychiatric Epidemiology Program, Michigan State University, East Lansing, Michigan, United States of America; Epilepsy Care Team, Chikankata Hospital, Mazabuka, Zambia; 5 Department of Addiction, National Addiction Centre, Institute of Psychiatry, King's College London, London, United Kingdom; 6 Cluster Manager, Non-communicable Diseases, National Department of Health, Pretoria, South Africa; 7 Department of Psychiatry, University Hospitals and Faculty of Medicine of the University of Geneva, Geneva; Department of Psychiatry, Division of Old Age Psychiatry, Hospices-CHUV, Lausanne, Switzerland; 8 Mental Health Services, Ministry of Health, Jerusalem, Israel; 9 Department of Psychology, University of Uyo, Uyo, Nigeria; 10 College of Medicine, University of Ibadan & University College Hospital, Ibadan, Nigeria; 11 Centre for Global Mental Health, London School of Hygiene & Tropical Medicine, United Kingdom; Sangath, Goa, India; 12 Shanghai Mental Health Center, Shanghai Jiao Tong University School of Medicine, Shanghai, China; Departments of Psychiatry and Global Health, Emory University School of Medicine, Atlanta, Georgia, United States of America; 13 King's College London, Institute of Psychiatry, Health Service and Population Research Department, London, United Kingdom; 14 Iranian Research Center for HIV/AIDS, Tehran University of Medical Sciences, Tehran, Iran; 15 University of Liverpool, Institute of Psychology, Health & Society; Child Mental Health Unit, Alder Hey Children's NHS Foundation Trust, Liverpool, United Kingdom; 16 UCL Institute of Neurology, Queen Square, London, United Kingdom, SEIN – Epilepsy Institute in the Netherlands Foundation, Heemstede, The Netherlands; 17 Faculty of Medicine, University of Sydney, Sydney, Australia; 18 Child and Adolescent Health and Development, WHO Eritrea, Asmara, Eritrea; 19 Schizophrenia Research Foundation (SCARF), Chennai, India; 20 Department of Psychiatry and Behavioural Sciences, University of Washington Medical Center; UW AIMS Center; IMPACT Implementation Program, Seattle, Washington, United States of America; 21 HealthNet TPO, Amsterdam, The Netherlands; 22 SNEHA and Voluntary Health Services, Chennai, India; 23 Health Service and Population Research Department, Institute of Psychiatry, King's College London, London, United Kingdom

## Abstract

Shekhar Saxena and colleagues summarize the recent WHO Mental Health Gap Action Programme (mhGAP) intervention guide that provides evidence-based management recommendations for mental, neurological, and substance use (MNS) disorders.

**Summary Points**

- The treatment gap for mental, neurological, and substance use (MNS) disorders is more than 75% in many low- and middle-income countries.
- In order to reduce the gap, the World Health Organization (WHO) has developed a model intervention guide within its Mental Health Gap Action Programme (mhGAP).
- The model intervention guide provides evidence-based recommendations developed with the Grading of Recommendations Assessment, Development and Evaluation (GRADE) methodology.
- This article presents the management recommendations for MNS disorders, with a link to the World Health Organization website where all the background material may be accessed.
- To our knowledge, this is a first exercise involving such an extensive and systematic evaluation of evidence in this area.

## Why Evidence-Based Guidelines Are Urgently Needed

Mental, neurological, and substance use (MNS) disorders are highly prevalent and are responsible for 14% of the global burden of disease expressed in disability-adjusted life years (DALYs) [1]. The resources that have been provided in countries to tackle the huge burden are insufficient, inequitably distributed, and inefficiently used, which results in a large majority of people with these disorders receiving no care at all [2]–[7]. Even when available, treatment and care often is neither evidence-based nor of high quality. The result is a large treatment gap, with more than 75% in many low- and middle-income countries (LAMIC).

In order to reduce the gap, the World Health Organization (WHO) launched the Mental Health Gap Action Programme (mhGAP) to scale up services for people with MNS disorders, especially in LAMIC [1]. One essential component of mhGAP is to develop management recommendations (guidelines) for MNS disorders identified as conditions of high priority. The priority conditions included are depression, psychosis, bipolar disorders, epilepsy, developmental and behavioural disorders in children and adolescents, dementia, alcohol use disorders, drug use disorders, and self-harm/suicide. The priority conditions were selected because they represent a large burden [8] in terms of mortality, morbidity, or disability, have high economic costs, and are often associated with violations of human rights.

Evidence suggests that explicit, evidence-based, cost-effective packages of interventions can improve the processes and outcomes of health care when appropriately implemented. A package approach is being considered for a wide variety of health challenges such as the packages of interventions for family planning, safe abortion care, and maternal, newborn, and child health [9]. The recent grand challenges in global mental health underline the fact that all interventions should be evidence-based to provide programme planners, clinicians, and policy-makers with effective care packages [10]. While examples of evidence-based packages of care for MNS disorders exist that focus mainly on delivery mechanisms, they did not systematically synthesize and appraise the evidence base for interventions [11]. WHO therefore established a Guideline Development Group (GDG) in 2008 to develop evidence-based recommendations that adhered to the Grading of Recommendations Assessment, Development and Evaluation (GRADE) principles for developing transparent, evidence-based WHO guidelines [12].

**Figure 1:**
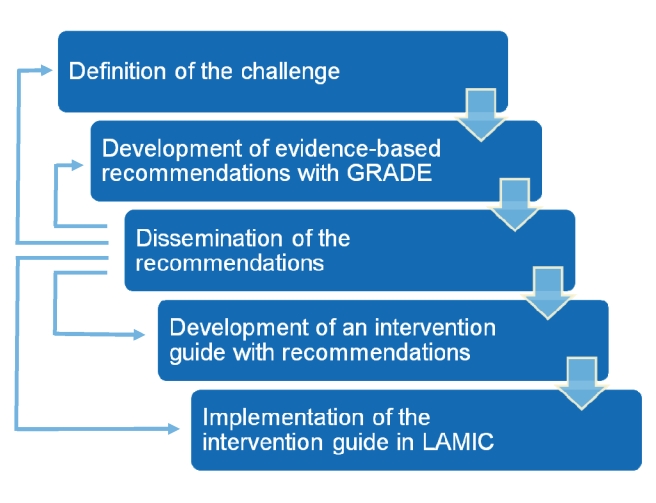
Steps in the formulation and implementation of the *mhGAP Intervention Guide*.

This article presents the management recommendations for MNS conditions, with a specific link (below) to the WHO website where all the background material may be accessed, including the evidence profiles and GRADE tables. Disseminating the recommendations through the scientific literature is important to generate feedback from health care professionals, policy–makers, and scientists. This will be helpful for re-defining the challenges (we may have omitted some important topics, or we should have addressed some other topics in a different way); re-evaluating the synthesized evidence; re-directing the development of the intervention guide for use in LAMIC; and re-defining any implementation strategies in LAMIC (Figure 1). In this article, we use the term “mental health” as a convenient label for MNS disorders.

## mhGAP Recommendations

WHO recommendations aim to be based on systematic reviews of the best available evidence and consideration of values, preferences, and feasibility issues from an international perspective and as reflected in relevant WHO policy documents and technical tools. Evidence profiles were developed for each of the priority conditions as well as a category labeled “other significant emotional or medically unexplained complaints.” Tables 1–8 provide the running title of the question and the abridged recommendations for each of the conditions. These recommendations focus on the management of MNS disorders in non-specialist settings in LAMIC. Each recommendation has a unique identifier—a short form of the priority condition and a number (e.g., DEP 1 for depression question 1). The detailed evidence profile can be accessed from the mhGAP Evidence Resource Centre (http://www.who.int/mental_health/mhgap/evidence/en/index.html).

**Table 1:** Abridged recommendations for depression (DEP 1–6) and other significant emotional or medically unexplained complaints (OTH 1–7).

**Role of antidepressants and benzodiazepines**	DEP 1. Antidepressants should not be considered for the initial treatment of adults with mild depressive episode. Tricyclic antidepressants (TCA) or fluoxetine should be considered in adults with moderate to severe depressive episode/disorder.OTH 2. Neither antidepressants nor benzodiazepines should be used for the initial treatment of individuals with complaints of depressive symptoms in absence of current/prior depressive episode/disorder.
**Duration of antidepressant treatment**	DEP 2. Antidepressant treatment should not be stopped before 9–12 months after recovery.
**Brief, structured, psychological treatment**	DEP 3. Interpersonal therapy and cognitive behavioural therapy (CBT) (including behavioural activation, DEP 4), and problem-solving treatment should be considered as psychological treatment of depressive episode/disorder in non-specialized health care settings if there are sufficient human resources (e.g., supervised community health workers). In moderate and severe depression, problem-solving treatment should be considered as adjunct treatment.OTH 3. A problem-solving approach should be considered in people with depressive symptoms (in the absence of depressive episode/disorder) who are in distress or have some degree of impaired functioning.OTH 1. Psychological treatment based on CBT principles should be considered in repeat adult help seekers with medically unexplained somatic complaints who are in substantial distress and who do not meet criteria for depressive episode/disorder.
**Relaxation training and physical activity**	DEP 5, DEP 6. Relaxation training and advice on physical activity may be considered as treatment of adults with depressive episode/disorder. In moderate and severe depression, these interventions should be considered as adjunct treatment.
**Psychological support after recent traumatic event**	OTH 4. Psychological debriefing should not be used for recent traumatic event to reduce the risk of post-traumatic stress, anxiety, or depressive symptoms.OTH 5. Providing access to support based on the principles of psychological first aid should be considered for people in acute distress exposed recently to a traumatic event.
**Graded self-exposure based on CBT principles in adults with post-traumatic stress disorder (PTSD) symptoms**	OTH 6. If it is possible to continue to follow up with the patient, graded self-exposure based on the principles of CBT should be considered in adults with PTSD symptoms.
**Psychological treatment based on CBT principles in people concerned about prior panic attacks**	OTH7. Psychological treatment based on CBT principles should be considered as treatment of people concerned about prior panic attacks.

**Table 2 pmed-1001122-t002:** Abridged recommendations for epilepsy and seizures (EPI 1–12).

**Management of acute convulsive seizures and status epilepticus**	EPI 1. Intravenous (IV) access not available: Rectal diazepam should be administered. Intramuscular (IM) administration of diazepam is not recommended because of erratic absorption. IM phenobarbital may also be considered when rectal use of diazepam is not possible due to medical or social reasons.EPI 2. IV access available: An IV benzodiazepine (lorazepam or diazepam) should be administered (if available, lorazepam is preferred over diazepam). For sustained control or if seizures continue, IV phenobarbital or phenytoin should be administered.
**Management of febrile seizures**	EPI 3. In simple febrile seizures, local standards for diagnosis and management of fever (including Integrated Management of Childhood Illnesses) should be followed and children should be observed for 24 hours. Children with complex febrile seizures (CFS) should be observed within an inpatient setting. Investigations such as blood tests and lumbar puncture to determine the presence of underlying etiology is recommended as appropriate.Prophylactic intermittent diazepam during febrile illness may be considered in the treatment of recurrent or prolonged CFS but not for simple febrile seizures.
**Diagnosis of convulsive epilepsy**	EPI 4. Non-specialist health care providers can be trained to recognize and diagnose convulsive epilepsy. Such training should be provided.EPI 5 & EPI 6. Electroencephalography (EEG) and neuroimaging should not be used routinely for diagnosis of epilepsy and starting treatment in non-specialized health care settings in LAMIC. If required for formulation of etiological diagnosis, EEG and neuroimaging should be done in specialized facilities under optimum technical conditions and with adequate expertise for interpretation of the data and results.
**Antiepileptic drugs**	EPI 7. In convulsive epilepsy, monotherapy with any of the standard antiepileptic drugs (carbamazepine, phenobarbital, phenytoin, and valproic acid) should be offered. Given the acquisition costs, phenobarbital should be offered as a first option if availability can be assured. If available, carbamazepine should be preferentially offered to children and adults with partial onset seizures.EPI 8. Antiepileptic drugs should not be routinely prescribed to adults and children after a first unprovoked seizure.EPI 9. Discontinuation of antiepileptic drug treatment should be considered after 2 seizure-free years. The decision to withdraw or continue antiepileptic drugs in a seizure-free patient should be made after consideration of relevant clinical, social, and personal factors and with the involvement the patient and the family.
**Psychological interventions**	EPI 10. Information and advice on avoiding high-risk activities and first aid relevant to the person and family members should be routinely given. Psychological treatments such as relaxation therapy, treatments based on cognitive behavioural therapy principles, psychoeducational programmes, and family counselling may be considered as adjunctive treatment.
**Management of epilepsy in women of childbearing age**	EPI 11. Women with epilepsy should have seizures controlled as well as possible with antiepileptic drug monotherapy at minimum effective dose. Valproic acid should be avoided if possible. Antiepileptic drug polytherapy should be avoided.Folic acid should routinely be taken when they are on antiepileptic drugs. Standard breast feeding recommendations remain appropriate for the antiepileptic drugs included in this review (phenobarbital, phenytoin, carbamazepine, and valproic acid).
**Management of epilepsy and associated intellectual disability**	EPI 12. People with intellectual disability and epilepsy should have access to the same range of investigations and treatment as the rest of the population. The drug of choice depends on the type of seizure and should be individualized. When available, consider either valproic acid or carbamazepine instead of phenytoin or phenobarbital due to lower risk of behavioural adverse effects.

CFS, complex febrile seizures; EEG, electroencephalography; IM, intramuscular; IV, intravenous.

**Table 3 pmed-1001122-t003:** Abridged recommendations for psychosis and bipolar disorders (PSY 1–13).

*Psychosis*	
**Role of antipsychotic medicines**	PSY 1. Haloperidol or chlorpromazine should be routinely offered. Second-generation antipsychotics (with the exception of clozapine) may be an alternative if availability can be assured and cost is not a constraint. For individuals who do not respond to antipsychotic medicines, clozapine may be considered by non-specialist health care providers, preferably under the supervision of mental health professionals, only if routine laboratory monitoring is available.
**Use of two or more antipsychotic medications concurrently**	PSY 2. Routinely, one antipsychotic should be prescribed at a time. For individuals who do not respond to antipsychotic medicines (using one medicine at a time), antipsychotic combination treatment may be considered by primary health care professionals, preferably under the supervision of mental health professionals with close clinical monitoring.
**Length of antipsychotic treatment in individuals with a first psychotic episode**	PSY 3. Antipsychotic treatment should be continued for at least 12 months after the beginning of remission.
**Antipsychotic withdrawal in individuals with long term and/or recurrent illness**	PSY 4. In individuals stable for several years on antipsychotic treatment, withdrawal may be considered keeping in mind the increased risk of relapse, possible adverse effects of medicines, and individual preferences in consultation with the family. This decision should be made preferably in consultation with a mental health professional.
**Role of depot antipsychotic medication**	PSY 5. Individuals on long-term antipsychotic treatment should be given adequate information and encouraged to make a choice between oral and depot preparations, especially with the view to improve adherence.
**Role of anticholinergic medications in preventing or reducing extrapyramidal side-effects**	PSY 6. Anticholinergics should not be used routinely for preventing extrapyramidal side effects. Short-term use may be considered only in individuals with significant extrapyramidal side effects when dose reduction and switching strategies have proven ineffective, or when these side effects are acute or severe.

*Bipolar disorder*	
**Role of medicines in the manic phase**	PSY 7. Haloperidol is recommended in individuals with bipolar mania. Second-generation antipsychotics may be considered as an alternative if availability can be assured and cost is not a constraint. Lithium, valproate, or carbamazepine should be offered to individuals with bipolar mania. Treatment with lithium should be initiated only in those settings where personnel and facilities for close clinical and laboratory monitoring are available.
**Role of medicines in the maintenance phase**	PSY 8. Lithium or valproate should be used for the maintenance treatment of bipolar disorder. Maintenance treatment should continue for at least 2 years after the last episode of bipolar disorder. Decision to continue maintenance treatment after 2 years should preferably be done by a mental health specialist.
**Role of medicines in the depressive phase**	PSY 9. Antidepressant medicines, always in combination with a mood stabilizer (lithium or valproate), may be considered in the treatment of moderate or severe depressive episodes of bipolar disorder. Selective serotonin reuptake inhibitors (SSRI; fluoxetine) should be preferred to tricyclic antidepressants (TCA).

*Psychosocial interventions*	
**Role of psychoeducation**	PSY 10. Psychoeducation should be routinely offered to individuals with psychotic and bipolar disorders and their family members/caregivers. Cognitive behavioural therapy and family interventions can be considered as an option if adequately trained professionals are available.
**Role of psychosocial strategies**	PSY 11. Psychosocial interventions to enhance independent living and social skills should be considered for people with psychotic and bipolar disorders and their families/caregivers. Social skills training may be considered as an option, in association with other psychosocial interventions. Facilitation of supported housing/assisted living facilities as well as independent living facilities may be considered as an option, with careful consideration to patients' functional capacity and need for stability and support.
**Role of vocational and economic inclusion**	PSY 12. Non-specialist health care providers should facilitate opportunities for people with severe mental, neurological, and substance use disorders and their families/caregivers to be included in economic activities appropriate to their social and cultural environment, using formal and non-formal systems that may be available. Facilitation of supported employment may be considered as an option if they have difficulty in obtaining or retaining normal employment.
**Role of strategies aimed at improving community attitudes**	PSY 13. Activities aimed at improving community attitudes towards people with mental, neurological, and substance use conditions (e.g., anti-stigma campaigns) may be considered for implementation.

**Table 4 pmed-1001122-t004:** Abridged recommendations for self-harm and suicide (SUI 1–9).

**Role of asking about thoughts, plan or act of self-harm**	SUI 1. Individuals over 10 years of age suffering from any of the other priority conditions, or who present with chronic pain or acute emotional distress associated with current interpersonal conflict, recent loss, or other severe life event, should be asked about thoughts or plans of self-harm in the last month or acts of self-harm in the last year at initial assessment and periodically as required.
**Role of removing means for self-harm/suicide from a person**	SUI 2. The individual, family, and relevant others should be advised to restrict access to the means for self-harm (e.g., pesticides and other toxic substances, medication, firearms) as long as the individual has thoughts, plans, or acts of self-harm.
**Role of contact for persons with thoughts, plans or acts of self-harm**	SUI 3. Regular contact (telephone contact, home visits, letter, contact card, brief intervention and contact) with the non-specialized health care provider is recommended for persons with acts of self-harm in the last year, and should be considered for persons with thoughts or plans of self-harm in the last month.
**Role of a problem solving approach**	SUI 4. A structured problem-solving approach should be considered as a treatment for persons with acts of self-harm in the last year, if there are sufficient human resources.
**Role of social support**	SUI 5. Use of social support (from available informal and/or formal community resources) should be facilitated for persons with thoughts or plans of self-harm in the last month or acts of self-harm in the last year.
**Role of hospitalization**	SUI 6. Hospitalization in non-specialized services of general hospitals with the goal of preventing acts of self-harm is not routinely recommended for persons with self-harm. If imminent risk of self-harm is a concern, urgent referral to a mental health service should be considered. However, if such a service is not available, family, friends, concerned individuals, and other available resources should be mobilized to ensure close monitoring.
**Role of reducing access to means of suicide at the population level**	SUI 7. Restricting access to means of self-harm (such as pesticides, firearms, high places) is recommended.
**Role of reducing the availability of alcohol**	SUI 8. Policies to reduce harmful use of alcohol should be developed as a component of a comprehensive suicide prevention strategy, particularly within populations with high prevalence of alcohol use.
**Role of responsible and deglamourized media reporting**	SUI 9. Responsible media reporting of suicide (such as avoiding language which sensationalizes or normalizes suicide or presents it as a solution to a problem, avoiding pictures and explicit description of the method used, and providing information about where to seek help) is recommended.

**Table 5 pmed-1001122-t005:** Abridged recommendations for dementia (DEM 1–10).

**Role of acetylcholinesterase inhibitors and memantine**	DEM 1 & DEM 2. Acetylcholinesterase inhibitors or memantine should not be considered routinely for people with dementia in non-specialist health settings in LAMIC. They may be considered where adequate support and supervision by specialists is available. Consideration should be given to adherence and monitoring of adverse effects, which generally requires the availability of a carer.
**Role of medicines including antipsychotics for behavioural and psychological symptoms of dementia**	DEM 3. Thioridazine, chlorpromazine, or trazodone should not be used for the treatment of behavioural and psychological symptoms of dementia.Haloperidol and atypical antipsychotics should not be used as first-line management. Where there is clear and imminent risk of harm with severe and distressing symptoms, their short-term use may be considered, preferably in consultation with specialist.
**Role of antidepressants**	DEM 4. In people with dementia with moderate or severe depression, use of selective serotonin reuptake inhibitors may be considered. In case of non-response after at least 3 weeks, they should preferably be referred to a mental health specialist for further assessment and management.
**Cognitive and psychosocial interventions**	DEM 5. Cognitive interventions applying principles of reality orientation, cognitive stimulation, and/or reminiscence therapy may be considered in the care of people with dementia. Health care providers should be trained for delivering these interventions and family members should be involved in delivery of these interventions.
**Diagnosis of dementia**	DEM 6. Non-specialist health care providers should seek to identify possible cases of dementia in the primary health care setting and in the community after appropriate training and awareness raising. Brief informant assessment and cognitive tests should be used to assist in confirming these cases. For a formal dementia diagnosis, a more detailed history, medical review, and mental state examination should be carried out to exclude other common causes of cognitive impairment and decline.DEM 7. People with dementia and their family members should be told of the diagnosis subject to their wishes in this regard, keeping in mind cultural sensitivities and employing some preparatory work to determine their preferences. It should be accompanied with relevant information appropriate to culture and understanding of people, and with a commitment of ongoing support and care that can be provided by health and other services.
**Role of a medical review**	DEM 8. People with dementia should receive an initial and a regular medical review (at least every 6 months) and appropriate care.
**Interventions for carers of people with dementia**	DEM 9. Psychoeducational interventions should be offered to family and other informal carers of people with dementia at the time when diagnosis is made. Training of carers involving active carer participation (e.g., role playing of behavioural problem management) may be indicated later in the course of illness for carers who are coping with behavioural symptoms in people with dementia. Carer psychological strain should be addressed with support, counselling, and/or cognitive behaviour interventions. Depression in carers should be managed according to the recommendations for depression.DEM 10. Where feasible, home-based respite care may be encouraged for carers of people with dementia.

**Table 6 pmed-1001122-t006:** Abridged recommendations for alcohol use disorders (ALC 1–6).

**Screening and brief interventions**	ALC 1. Screening for hazardous and harmful alcohol use should be conducted, using a validated instrument that can be easily incorporated into routine clinical practice (e.g., AUDIT-3, AUDIT-C, ASSIST). In settings in which screening is not feasible or affordable, practitioners should explore alcohol consumption in their patients when relevant.Patients with a hazardous and harmful alcohol use should receive a brief intervention.Patients who on screening are identified as having dependence should be managed according to the recommendations in the section on alcohol dependence.
**Management of alcohol withdrawal**	ALC 2 & ALC 3. Supported withdrawal from alcohol should be advised in patients with alcohol dependence.Benzodiazepines are recommended as front-line medication for the management of alcohol withdrawal in alleviating withdrawal discomfort, and preventing and treating seizures and delirium. Antipsychotic medications should not be used as stand-alone medications for the management of alcohol withdrawal. They should only be used as an adjunct to benzodiazepines in severe withdrawal delirium that has not responded to adequate doses of benzodiazepines.Anticonvulsants should not be used following an alcohol withdrawal seizure for the prevention of further alcohol withdrawal seizures.Psychoactive medication used for the treatment of alcohol withdrawal should be dispensed in small quantities, or each dose supervised, to reduce the risk of misuse.Patients at risk of severe withdrawal, or who have concurrent serious physical or psychiatric disorders, or who lack adequate support, should preferably be managed in an inpatient setting.As part of withdrawal management, all patients should be given oral thiamine. Patients at high risk (malnourished, severe withdrawal) or with suspected Wernicke's encephalopathy should be given parental thiamine.
**Preventing relapse in alcohol dependent patients**	ALC 4. Acamprosate, disulfiram, or naltrexone should be offered as part of treatment to reduce relapse in alcohol dependent patients. The decision to use acamprosate, disulfiram, or naltrexone should be made taking into consideration patient preferences, motivation, and availability.
**Psychosocial interventions for management of alcohol dependence**	ALC 5. Psychosocial support should be routinely offered to alcohol dependent patients. Where providers have capacity, more structured psychological interventions, such as motivational techniques, should be considered.Non-specialist health care providers should consider involving family members in the treatment of the patient with alcohol dependence, where appropriate, and offer support to family members in their own right.
**Role of mutual help groups such as Alcoholics Anonymous (AA)**	ALC 6. Non-specialist health care workers should familiarize themselves with locally available mutual help groups (such as Alcoholics Anonymous), and should encourage the alcohol dependent patient to engage with such a group. They should monitor the impact of attending the group on the patient with alcohol dependence.Family members of patients with alcohol dependence should also be encouraged to engage with an appropriate mutual help group for families.

**Table 7 pmed-1001122-t007:** Abridged recommendations for drug use disorders (DRU 1–6).

**Brief psychosocial interventions**	DRU 1. Individuals using cannabis and psychostimulants should be offered brief intervention, which should comprise a single session of 5–30 minutes in duration, incorporating individualized feedback and advice on reducing or stopping cannabis/psychostimulant consumption, and the offer of follow up.People with ongoing problems related to their cannabis or psychostimulant drug use who do not respond to brief interventions should be considered for referral for specialist assessment.
**Management of drug withdrawal**	DRU 2. Withdrawal from cannabis, cocaine, or amphetamines is best undertaken in a supportive environment. No specific medication is recommended for the treatment of their withdrawal.Relief of symptoms (e.g., agitation, sleep disturbance) may be achieved with symptomatic medication for the period of the withdrawal syndrome. Less commonly, depression or psychosis can occur during withdrawal; in these cases the individual needs to be monitored closely and advice sought from relevant specialists, if available.Withdrawal from benzodiazepines is best undertaken in a planned (elective) manner, using a gradually tapering dose over 8–12 weeks and with conversion to long-acting benzodiazepines, rather than using short-acting ones. Additional psychosocial support should be considered. If a severe benzodiazepine withdrawal syndrome develops, specialist advice should be obtained regarding starting a high-dose benzodiazepine sedation regime and hospitalization.
**Treatment of psychostimulant dependence**	DRU 3. Dexamphetamine should not be offered for the treatment of stimulant use disorders.
**Psychosocial support for the management of cannabis dependence and abuse and psychostimulant use disorders**	DRU 4 & DRU 5. Short duration psychosocial support modeled on motivational principles should be offered for the treatment of cannabis use disorders and psychostimulant use disorders in non-specialized settings.Individuals with these disorders who do not respond to short duration psychological support may be referred for treatment in a specialist setting, when available.
**Role of sterile injection equipment and outreach programmes for injecting drug users**	DRU 6. In communities with a high prevalence of drug injection, primary health care providers should facilitate the provision of sterile injection equipment and retrieval of used equipment in primary care centres through involvement of community pharmacies or through outreach programmes.If resources allow, outreach programmes should be implemented to facilitate access to sterile injecting equipment (and retrieval), information, health care (including testing and counseling for HIV, and hepatitis), and entry to drug treatment.

**Table 8:** Abridged recommendations for child and adolescent mental health conditions (CAMH 1–13).

**Maternal mental health interventions**	CAMH 1. For at-risk children, parenting interventions promoting mother–infant interactions, including psychosocial stimulation, should be offered to improve child development outcomes. To improve child development outcomes, mothers with depression or with any other mental, neurological or, substance use condition should be treated using effective interventions (see recommendations for treatment of depression and other mental, neurological, or substance use conditions).
**Parent skills training for behavioural disorders**	CAMH 5. Parent skills training should be considered for the treatment of emotional and behavioural disorders in children aged 0–7 years. The content should be culture sensitive but should not allow violation of children's basic human rights according to internationally endorsed principles.
**Parent skills training for developmental disorders**	CAMH 6. Parent skills training should be considered in the management of children with intellectual disabilities and pervasive developmental disorders (including autism). Such training should use culturally appropriate training material.
**Child abuse**	CAMH 2. Non-specialized health care facilities should consider home visiting and offer parent education to prevent child abuse, especially among at-risk individuals and families. They should also collaborate with school-based “sexual abuse prevention” programmes where available.
**Intellectual disabilities**	CAMH 3. Non-specialized health care providers should consider assessment and regular monitoring of children suspected of intellectual and other developmental delays by brief, locally validated questionnaires. Clinical assessment under the supervision of specialists to identify common causes of these conditions should be considered.CAMH 4. Non-specialized health care providers should consider supporting, collaborating with, and facilitating referral to and from community-based rehabilitation programmes.
**Behaviour disorders (attention deficit hyperactivity disorder [ADHD])**	CAMH 7. Non-specialized health care providers at the secondary level should consider initiating parent education/training before starting medication for a child who has been diagnosed as suffering from ADHD. Initial interventions may include cognitive behavioural therapy (CBT) and social skills training if feasible.Methylphenidate may be considered, when available, after a careful assessment of the child, preferably in consultation with the relevant specialist and taking into consideration the preferences of parents and children.
**Pharmacological interventions for children with disruptive behaviour disorders or conduct disorder or oppositional defiant disorder**	CAMH 8. Pharmacological interventions (such as methylphenidate, lithium, carbamazepine, and risperidone) should not be offered by non-specialized health care providers to treat disruptive behaviour disorders (DBD), conduct disorder (CD), oppositional defiant disorder (ODD), and comorbid ADHD. For these conditions, the patients should be referred to a specialist before prescribing any medicines.
**Somatoform disorders**	CAMH 9. Pharmacological interventions should not be considered by non-specialized health care providers. Brief psychological interventions, including CBT, should be considered to treat somatoform disorders in children, if adequate training and supervision by specialists can be made available.
**Antidepressants for children with depression**	CAMH 10. Antidepressants should not be used for the treatment of children 6–12 years of age with depressive episode/disorder in non-specialist settings.
**Antidepressants for adolescents with depression**	CAMH 11. Fluoxetine, but not tricyclic antidepressants (TCA) or other selective serotonin reuptake inhibitors (SSRI), may be considered as one possible treatment in non-specialist settings of adolescents with depressive episodes. Adolescents on fluoxetine should be monitored closely for suicide ideas/behaviour. Support and supervision from a mental health specialist should be obtained, if available.
**Pharmacological interventions for anxiety disorders in children and adolescents**	CAMH 12. Pharmacological interventions should not be considered in children and adolescents with anxiety disorders in non-specialist settings.
**Behaviour change techniques for promoting mental health**	CAMH 13. Non-specialized health care facilities should encourage and collaborate with school-based life skills education, if feasible, to promote mental health in children and adolescents.

## Strengths and Limitations of the Process

Developing recommendations is a complex process that involves systematic review and assessment of the quality of evidence and balance of benefits and harms. In addition, explicit consideration of other issues such as value judgments, resource use, and feasibility, which are major considerations, need to be incorporated. Developing mhGAP recommendations within this framework highlighted several challenging and critical issues, including difficulties in formulating questions and rating outcomes, potential reproducibility and consistency problems, problems in incorporating observational evidence when evidence in the form of randomized trials was not available, and difficulties in defining how values, preferences, and feasibility issues should be considered, as the methodology for these aspects is much less developed [12].

The mhGAP guidelines highlight issues where uncertainty or controversy exists or areas where a change in policy or clinical practice is needed. Table 9 provides examples of some areas where the review of evidence, values, and resource considerations supported the change required in clinical practice.

**Table 9:** Clinical practice implications of mhGAP recommendations.

Clinical Practice	Evidence	mhGAP Recommendation
Patients with depressive symptoms, but without moderate or severe depression, are frequently treated with antidepressants.	In people with depressive symptoms (in absence of depressive episode/disorder), there is unlikely a clinically important difference between antidepressants and placebo [23]. In people with current depressive episode/disorder, available evidence suggests that the drug–placebo differences for antidepressants increase as a function of initial severity, rising from virtually no difference in mild depression to a small difference for adults with moderate depression and a medium difference in severe depression [24].	Antidepressants should not be considered for the initial treatment of adults with depressive symptoms in absence of current/prior moderate or severe depressive episode/disorder.
Combination of antipsychotic and anticholinergic medicines is commonly used.	There is no evidence supporting routine use of anticholinergic drugs [25].	Anticholinergics should not be used routinely for preventing extrapyramidal side effects.
EEG is commonly used to confirm or exclude diagnosis of epilepsy in individuals with suspected seizures.	EEG is a diagnostic test with variable sensitivity and specificity [26]. It may be normal in people with epilepsy and can be abnormal in normal people. In addition, technical and interpretative standards need to be met.Overinterpretation of EEG and misdiagnosis of epilepsy is an important problem [27],[28].	EEG and neuroimaging should not be used routinely for diagnosis of epilepsy and starting treatment in non-specialized health care settings in low and middle income countries.
Treatment of alcohol dependence has, in many settings, been considered something that needs the support of a specialist treatment service. Many primary care services are reluctant to get involved in the treatment of alcohol dependence themselves.	Medications such as acamprosate and naltrexone are effective in primary care and result in significantly improved treatment outcomes. Disulfiram is also effective, and can also be used in primary care, with specialist support if needed [29].	Acamprosate, disulfiram, or naltrexone should be offered as part of treatment to reduce relapse in alcohol dependent patients. The decision to use acamprosate, disulfiram, or naltrexone should be made taking into consideration patient preferences, motivation, and availability.
Inappropriate diagnosis of ADHD and use of stimulants have often been described, raising concerns when the treatment is offered by non-specialized health providers.	Parent training, cognitive-behavioural therapy, and social skills have been shown to be effective for ADHD [30],[31]. Methylphenidate has been shown to be effective for reducing hyperactivity and improving quality of life. And despite treatment acceptability limitations and side effects, it has been recommended for treating ADHD [30]–[32].	Non-specialized health care providers at the secondary level should consider initiating parent education/training before starting medication for a child who has been diagnosed as suffering from ADHD. Initial interventions may include CBT and social skills training if feasible.Methylphenidate may be considered, when available, after a careful assessment of the child, preferably in consultation with the relevant specialist and taking into consideration the preferences of parents and children.
Off-label prescribing of antipsychotic drugs has been commonly employed to treat symptoms of aggression, agitation, and psychosis in patients with dementia.	The evidence review suggests that these drugs appear to have only a limited positive effect in treating these symptoms but can cause significant harm to people with dementia [33].	Thioridazine or chlorpromazine should not be used for the treatment of behavioural and psychological symptoms of dementia. Haloperidol and atypical antipsychotics should not be used as first line management. Where there is clear and imminent risk of harm with severe and distressing symptoms, their short-term use may be considered, preferably in consultation with a specialist.
Psychiatric or psychological assessment or help besides the treatment of somatic symptoms does not include systematic assessment of suicide ideas.	Evidence of priority conditions, chronic pain, and emotional distress being associated with suicide. Evidence that asking about suicide ideas or thoughts does not increase the risk of committing suicide [34],[35].	Persons suffering from any of the other priority conditions or who present with chronic pain or acute emotional distress should be asked about thoughts, plans, or acts of self-harm.
Specialist services for drug use disorders are difficult to access in many countries. Non-specialists often do not feel confident to discuss drug problems with drug users.	Brief psychosocial interventions are effective in reducing cannabis and psychostimulant use. These brief interventions can be delivered by non-specialists by engaging the person using drugs in a short structured discussion incorporating motivational principles [36].	Short duration psychosocial support modeled on motivational principles should be offered for the treatment of cannabis use disorders and psychostimulant use disorders in non-specialized settings.

One of the most challenging aspects of the whole process was that while for some mental health areas the background evidence was quite robust, for others the conventional way of synthesizing and evaluating the evidence revealed no or very poor quality evidence that was insufficient to make any recommendation. For example, some psychosocial interventions for individuals with psychosis or bipolar disorder are backed by few studies that provide inconclusive results. In such instances we took advantage of the GRADE methodology, which clearly recognizes that in addition to the evidence base, other aspects that are expected to inform the recommendations include consideration of values such as protection of human rights, feasibility and resource use, and GDG professional experience and their tacit knowledge. As previously noted, the added value of GRADE in these circumstances is that it requires GDG to transparently report that some recommendations are based on strong values and weak evidence [12]. By contrast, in other areas where a substantial amount of evidence is available, positive recommendations could not be issued due to considerations on feasibility aspects. For example, full cognitive behavioural therapy may be hardly feasible in many low- and middle-income settings considering the training, supervision, and time needed for this intervention, and taking into consideration the need for local adaptations. Similarly, drug treatments that require regular blood checks may not be recommended in settings where no infrastructure is available to provide such checks on a continuous basis.

Another challenging aspect was that while the main focus of WHO's mental health work was to develop recommendations for health care providers in low- and middle-income settings, most of the evidence comes from specialist settings of high-income countries. Despite this, we developed and applied instructions to overcome this compelling issue [12]; increasing implementation research in the contexts where the guidelines should eventually be applied would seem the best approach to enhance the applicability of research findings into practice.

## Maintaining and Updating mhGAP Recommendations

Maintaining and updating evidence-based recommendations is similarly relevant, as new evidence is continuously generated and accumulated in the international literature. Updating of mhGAP recommendations will not only be based on this new evidence, but also on feedback from health care professionals, policy-makers, scientists, and public health experts involved in implementing the guidelines. We also expect that revisions might include review of new questions or areas currently not covered by mhGAP guidelines, which might be identified as relevant questions to be addressed. In order to facilitate and encourage feedback, an electronically accessible web repository of recommendations and background material, including all the evidence profiles that were used to generate the recommendations, has recently been developed and will be regularly maintained and updated (http://www.who.int/mental_health/mhgap/evidence/en/index.html). The readers of this article can also provide their feedback by posting comments online at *PLoS Medicine*. WHO will follow a systematic procedure for updating the recommendations following the same strict and transparent methodological process that was employed for their initial development.

## Guideline Implementation Issues

Scaling up delivery of interventions and achieving a desirable level of mental health care quality requires a dynamic and collaborative approach. Research findings that are generated by studies and trials should guide health care providers when making decisions in clinical practice. However, it is very difficult for the individual health care provider to interpret the existing evidence coming from randomized controlled trials, observational studies, and other sources of scientific evidence. Even if the evidence is systematically reviewed, the translation into the relevant policy and programme decisions, as well as the implementation in practice, is not straightforward. Effective strategies, techniques, and incentives are needed to disseminate and implement the guidelines. Finally, the interventions need to be adopted and integrated into practice by providers at all levels [13].

In the field of mental health care, the issue of how guidelines should be implemented to maximize their impact on clinician performance and patient outcome has rarely been investigated. A recent meta-analysis of different guideline implementation strategies included only 18 studies, nine of which were randomized trials [14]. The analysis of these 18 studies revealed that multifaceted interventions were more likely to have an impact on clinician performance and patient outcome, albeit effect sizes were generally modest. Additionally, it has been shown that audit of clinical activities and feedback to doctors may be a relevant component of any implementation strategy [15]. These findings are in line with those of a landmark systematic review of implementation studies [16].

The scarce evidence base on how guidelines should be implemented must not weaken international efforts to develop and evaluate implementation plans [17]. As part of the WHO implementation strategy, mhGAP recommendations have been incorporated into an integrated package of interventions called *mhGAP Intervention Guide* (mhGAP-IG) *for Mental, Neurological and Substance Use Disorders in Non-specialized Health Settings* [18]. The mhGAP-IG translates the evidence-based recommendations into simple clinical protocols and algorithms to facilitate decision making for assessment and management. It is aimed at non-specialist health care providers working at first- and second-level facilities. It is important that they are trained and then supervised and supported by the specialists.

The mhGAP-IG provides an integrated package of interventions for the management of priority MNS disorders. The reasons for integrated packages are the following: one, packages are an effective and efficient way of improving health service delivery. Two, delivery of interventions as a package is relatively cost-effective in terms of training and supervision. Many interventions and conditions go naturally together because they can be delivered by the same person at different times and because a limited range of interventions are applicable to a variety of conditions. Three, packages help concentrate scarce resources on the services that provide the best “value for the money”. We believe that the implementation of mhGAP guidelines and scaling them up would lead to a decrease in the burden of MNS disorders. For example, 7 million DALYs (11% of burden due to depression) are avertable with 50% coverage with interventions for depression (anti-depressant drugs [generic TCAs or SSRIs] plus brief psychotherapy) [19]. Similarly, 23% of burden due to epilepsy is avertable at 50% coverage with standard antiepileptic drugs [20]. Thus, the gains that are possible from scaling up of the integrated package could be substantial.

Although the mhGAP-IG is the principal clinical tool being used as part of the scaling up strategy of the mhGAP program in countries, accompanying training materials are being developed for capacity building. Program implementation using the mhGAP-IG has already started in four countries: Ethiopia, Jordan, Nigeria, and Panama [21]. There are a number of major implementation research questions that now need addressing. mhGAP has provided a definitive platform to define “what” should be scaled up. The “how” question needs answering now. An example of such an initiative is the multi-partner PRogramme for Improving Mental health carE (PRIME) Project, which has the objective to generate evidence on the implementation and scaling up of treatment programmes for priority mental disorders in low-resource settings [22]. In the near future, further efforts should be made to introduce formal evaluations of the capability of these programs to induce relevant and persistent changes, and to generate useful insights on how implementation in LAMIC should be conducted to maximize benefit at sustainable costs.

**Box 1. Key mhGAP Implementation Research Areas**

Although research in the field of mental, neurological, and substance use disorders has significantly advanced in recent years, most of these advancements have been driven by the needs of health systems in the richest countries. To appropriately translate research findings into clinical and public health practices, it is critical to accelerate implementation research to evaluate interventions beyond the controlled conditions of research settings, and in the type of populations that suffer the largest proportion of the global burden of morbidity and mortality.

Research areas that will help in scaling up mhGAP coverage include:

1. Strengthening human resources, including capacity development and task shifting issues
2. Improving health financing to avoid out-of-pocket and point-of-care payments for health care
3. Simplifying interventions so that they can be delivered with fewer resources
4. Identifying intervention delivery channels for greater reach and equity, and lower costs, including engagement of the non-government sector
5. Improving programme management and synergies between mental health and other programmes such as maternal or child health to overcome barriers to effective programme implementation
6. Mobilizing community resources for improved intervention delivery and increased health care utilization

Some examples of research questions are:

*How can mental health care using mhGAP be “added upon” the existing maternal health programmes?*
*What are the specific barriers that hinder access to effective mental health interventions in rural populations?*
*How can we use electronic and mobile technology to improve follow up for people with mental, neurological, and substance use disorders?*
*How can the coverage, quality, and impact of mhGAP interventions improved by involving community health workers?*
*What is the cost to the health system of scaling up mhGAP interventions when delivered through routine primary health care?*

## Abbreviations

ADHD: attention deficit hyperactivity disorder
CBT: cognitive behavioural therapy
GRADE: Grading of Recommendations Assessment, Development and Evaluation
LAMIC: low- and middle-income countries
mhGAP: Mental Health Gap Action Programme
mhGAP-IG: *mhGAP Intervention Guide*
MNS: mental, neurological, and substance use
WHO: World Health Organization
